# Nocturnal respiratory abnormalities among ward-level postoperative patients as detected by the Capnostream 20p monitor: A blinded observational study

**DOI:** 10.1371/journal.pone.0280436

**Published:** 2023-01-20

**Authors:** Vichaya Champreeda, Raymond Hu, Brandon Chan, Owen Tomasek, Yuan-Hong Lin, Laurence Weinberg, Will Howard, Chong O. Tan

**Affiliations:** 1 Department of Anesthesia, Austin Health, Heidelberg, Victoria, Australia; 2 Department of Critical Care, Melbourne Medical School, The University of Melbourne, Victoria, Australia; Brigham and Women’s Hospital and Harvard Medical School, UNITED STATES

## Abstract

**Purpose:**

This prospective observational study aimed to establish the frequency of postoperative nocturnal respiratory abnormalities among patients undergoing major surgery who received ward-level care. These abnormalities may have implications for postoperative pulmonary complications (PPCs).

**Methods:**

Eligible patients underwent blinded noninvasive continuous capnography with pulse oximetry using the Capnostream^™^ 20p monitor over the first postoperative night. All patients received oxygen supplementation and patient-controlled opioid analgesia. The primary outcome was the number of prolonged apnea events (PAEs), defined as end-tidal carbon dioxide (EtCO_2_) ≤5 mmHg for 30–120 seconds or EtCO2 ≤5 mmHg for >120 seconds with oxygen saturation (SpO_2_) <85%. Secondary outcomes were the proportion of recorded time that physiological indices were aberrant, including the apnea index (AI), oxygen desaturation index (ODI), integrated pulmonary index (IPI), and SpO_2_. Exploratory analysis was conducted to assess the associations between PAEs, PPCs, and pre-defined factors.

**Results:**

Among 125 patients who had sufficient data for analysis, a total of 1800 PAEs occurred in 67 (53.4%) patients. The highest quartile accounted for 89.1% of all events. Amongst patients who experienced any PAEs, the median (IQR) number of PAE/patient was four (2–12). As proportions of recorded time (median (IQR)), AI, ODI, and IPI were aberrant for 12.4% (0–43.2%), 19.1% (2.0–57.1%), and 11.5% (3.1–33.3%) respectively. Only age, ARISCAT, and opioid consumption/kg were associated with PPCs.

**Conclusions:**

PAE and aberrant indices were frequently detected on the first postoperative night. However, they did not correlate with PPCs. Future research should investigate the significance of detected aberrations.

## Background

The immediate postoperative period is a high-risk period for respiratory abnormalities in the surgical patient. Respiratory abnormalities can manifest as changes in respiratory rate, apnea, or oxygen desaturation. Such abnormalities are anticipated in the presence of postoperative pulmonary complications (PPCs) but can reasonably be expected to occur even in the absence of established PPCs.

PPCs are a heterogeneous group of conditions that range from minor respiratory derangements, such as hypoxia and atelectasis, to more major disease states, such as pneumonia and respiratory failure [1]. PPCs are associated with significant postoperative morbidity and mortality [2]. Early detection of respiratory abnormalities could offer opportunities for intervention before more serious PPCs manifest. However, the frequency with which early postoperative respiratory abnormalities occur and whether these respiratory abnormalities are associated with the subsequent development of PPCs is unknown.

Postoperative respiratory events may develop due to various insults on respiratory control and mechanics by surgery, anesthesia, and opioid analgesics [3–7]. Opioid-induced respiratory depression occurs relatively frequently within the first 24 hours [8, 9]. Deaths from these events are frequently reported at night when patients are asleep and vulnerable to respiratory abnormalities, and nursing observations are typically infrequent [8]. Further, in those with prior sleep-disordered breathing, existing derangements can worsen in the context of sleep, residual anesthetic agents, and opioid use or the disruption of the sleep architecture after surgery [10]. While hypoxia, bradypnea, and apnea commonly occur [11–16], their clinical significance is unknown.

Recent studies using continuous oximetry or capnography have shown that intermittent nursing observation misses a large proportion of postoperative respiratory aberrations [11, 17–19]. Patients are also normally aroused when vital signs are taken, thereby obliterating respiratory abnormalities present during sleep. The ability to continuously monitor and assess ventilation and oxygenation without arousal could offer insight into respiration in the postoperative context. Continuous capnography overcomes the existing limitations of intermittent nursing monitoring for surgical patients. Continuous capnography has been used effectively to detect apnea and respiratory depression in many settings [17, 20–22]. Most modern capnography monitors are mobile and can be coupled with algorithms that integrate values from other sources, such as continuous oximetry, to provide real-time indices that can act as warning systems to help guide clinical decision-making [17, 23]. The Capnostream^™^ 20p monitor (Medtronic, Minneapolis, MN, USA) is one such monitor; it combines continuous capnography with continuous oximetry to provide information on the respiratory rate and oxygen saturation by pulse oximetry (SpO_2_) while also deriving other information from algorithmic programming, such as the apnea index (AI), oxygen desaturation index (ODI), and integrated pulmonary index (IPI). AI and ODI are the average number of apnea and oxygen desaturation events per hour, respectively. Apnea is defined as the absence of respiratory activity for ≥10 seconds, and ODI is defined as the number of times that the oxygen saturation drops by ≥4% from baseline within a 240 second period. The IPI integrates the respiratory rate, pulse rate, oxygen saturation, and end-tidal carbon dioxide measurements using fuzzy logic to provide a real-time global assessment of the patient’s cardiorespiratory status, where 10 reflects normal physiology, and 1 is the most deranged [24].

The primary aim of the present study was to establish the frequency of prolonged apnea events during the first postoperative night using the Capnostream^™^ 20p monitor (hereafter “the Capnostream monitor”) in a cohort of patients undergoing major surgery who received standard ward-level care. The secondary aims were to 1) describe the duration of other deranged physiological parameters in the study population, as measured by the Capnostream monitor; 2) describe the frequency of PPCs in the study population; and 3) explore the associations of monitoring-derived variables with the development of PPCs.

## Methods

### Study design

This was a prospective observational study.

### Study participants

The study was approved by Austin Health Human Research Ethics Committee (HREC/16/Austin/269) on 17 August 2016. A consent waiver was granted for this observational study, as no change was made to existing patient care. Data collection took place at the Austin Hospital and the Heidelberg Repatriation Hospital from August 2017 to January 2020.

Eligible patients were over 18 years of age undergoing either elective hip or knee replacement surgery, intra-abdominal surgery, or bariatric surgery who were scheduled to receive patient-controlled analgesia (PCA). As per local hospital policy, oxygen supplementation via nasal cannula is part of the nursing practice for the use of PCA, and continued for the duration of PCA use. After cessation of PCA, as per local policy, use of oxygen therapy is guided to maintain target SpO _2_>95%, unless altered criteria have been documented. Exclusion criteria were: 1) inability to tolerate nasal oxygen cannula; 2) postoperative critical care admission; or 3) unavailability of equipment or investigators. At our institution, the Capnostream monitor is prioritized for patients clinically requiring close respiratory monitoring. Therefore, if the monitor was not being used for clinical purposes, it was made available for the study.

### Data collection

Eligible patients were connected to the Capnostream monitor before the start of the first postoperative night and the monitor was connected to a continuous power source. This involved applying the proprietary nasal cannula, which provided oxygen supplementation while also allowing for continuous real-time recordings of end-tidal carbon dioxide (EtCO_2_) and the respiratory rate. The Nellcor MAXA pulse oximeter (Covidien, Mansfield, MA, USA) was also applied to provide continuous recordings of the SpO_2_ and pulse rate. The oxygen flow rate was adjusted to between 1–4 L/min. All monitor values were covered, and alarms were silenced to allow the study to be conducted in an observational manner without changing patient care.

All electronic monitoring data were captured at two-second intervals on the Capnostream monitor, and recording was commenced on a portal storage device after setup. Data were deidentified and extracted onto a Microsoft Excel spreadsheet (Microsoft Corp, Redmond, WA, USA) and stored on a secure server. Information on the patients’ demographics, operative data, anesthetics, medical history, ARISCAT score [25], PPCs, and other adverse outcomes (unplanned admission to the intensive care unit (ICU), length of stay in hospital, and mortality within 30 days) was extracted from the electronic medical records.

S1 Appendix includes further details of the data recorded.

We selected a priori the period between 2200 and 0600 hours as our window of observation because this was the period in which patients were likely to be asleep and experience adverse respiratory events, and monitoring by nursing staff was typically infrequent. Our pre-planned analysis only included patients with >90% complete continuous data. Patients missing data for more than 10% of the monitoring period were excluded from the final analysis.

### Outcome measures

The primary outcome measure was the frequency of prolonged apnea events (PAEs). These events were defined by either 1) EtCO_2_ ≤ 5 mmHg for 30–120 seconds or 2) EtCO_2_ ≤5 mmHg for >120 seconds, in association with SpO_2_ ≤ 85%, measured as the number of PAEs per recorded time for each patient [15, 26, 27]. Events where EtCO_2_ was ≤5mmg for >120 seconds but *not* associated with SpO_2_ ≤ 85% were deemed to be spurious readings.

The secondary outcome measures were the proportion of recorded time of deranged physiological parameters, as displayed by the Capnostream monitor, and the frequency of PPCs within seven days of surgery in the study population. The following metrics were used to define the deranged physiological parameters displayed by the Capnostream monitor: 1) AI ≥5 events/hour, 2) ODI ≥5 events /hour, 3) IPI ≤7, 4) IPI ≤4, and 5) SpO_2_ <90%. The threshold of ≥5 for AI was chosen as a surrogate for the apnea-hypopnea index of ≥5, which defines mild OSA [28], and the threshold of ≥5 for ODI was chosen because this cut off has reasonable sensitivity for the presence of OSA [29]. The thresholds of deranged IPI were selected based on the recommendation that a value ≤7 requires attention, and a value ≤4 requires intervention [24]. PPCs were defined as prolonged oxygen requirement, atelectasis, pneumonia, pleural effusion, or bronchospasm [2, 25].

### Data analysis

All analysis was conducted on a per-protocol basis. Demographic, operative, and anesthetic characteristics were presented with descriptive statistics. Numerical data were reported either as mean ± standard deviation or median and interquartile range (IQR) after testing for normality using the Kolmogorov-Smirnov test. Categorical data were presented as the number and percentage of the population.

The rates of PAEs and PPCs were reported as the number of events per reporting period. The duration of deranged AI, ODI, IPI, and SpO_2_ was reported as an average, expressed as minutes per hour of available monitoring. Frequency analysis was conducted, and the results were graphically represented as incidence curves.

Multivariable logistics analysis was conducted to explore the associations for the development of PPCs. Individual associated factors were first entered into a univariate binary logistic regression model with PPCs as the outcome of interest and were provisionally noted if the statistical testing indicated a *p*-value <0.20 (S1 Appendix). These associations were subsequently entered into a multivariable logistic regression model with variables dropped in a stepwise fashion using a log likelihood-ratio test. The final model was assessed for discrimination using receiver operating characteristic (ROC) curve analysis [30], and goodness-of-fit was tested using the Hosmer–Lemeshow test. The statistical significance was set at p = 0.05 for the final model. Data analysis was conducted using Stata version 16 (StataCorp, College Station, TX).

## Results

**Fig 1:**
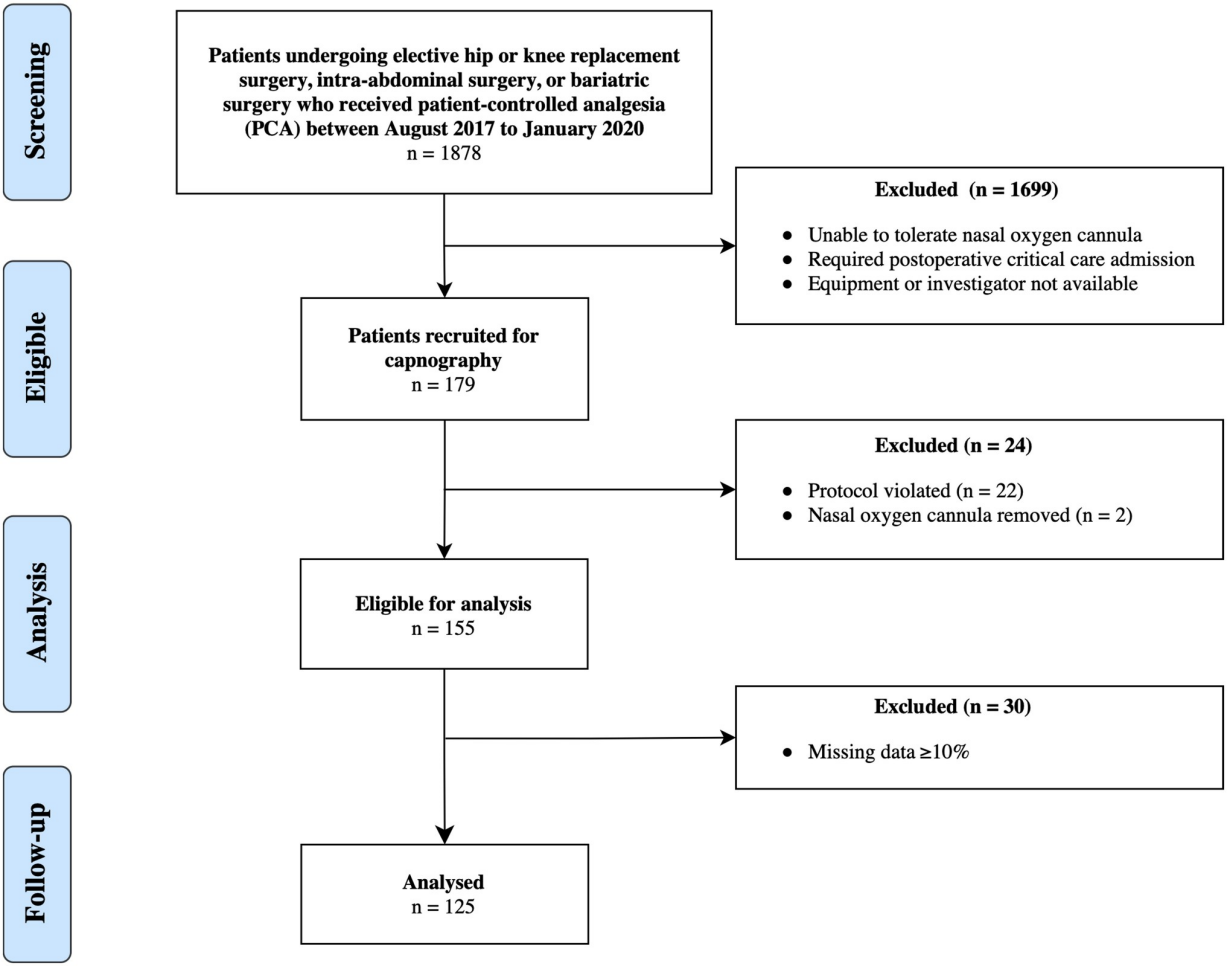
Patient flow diagram. Flow of patients through research study from screening to analysis.

A total of 179 patients were recruited for capnography performed on the first postoperative night out of a total of 1,878 patients undergoing eligible operations (Fig 1). One-hundred fifty-five patients were eligible for data analysis. As per protocol, 30 patients were excluded from the final analysis due to incomplete data recording (i.e. >10% missing). Therefore, analysis was conducted on the 125 patients who had sufficient data. Demographic data, comorbidities, and surgical and anesthetic characteristics are summarized in Table 1. The mean age was 59.7 ± 15.4 years. Forty-six patients (34.4%) were male, and the mean weight was 90.5 ± 23.7 kg. Nine patients (7.2%) had cardiac disease, and 23 patients (18.4%) had pulmonary diseases. Thirty-three (26.4%) patients were at high-risk of suffering from obstructive sleep apnea (OSA); however, there was no OSA risk assessment performed in 28 (22.4%) patients. The median ASA score was two (IQR 2–3). The median ARISCAT score was 19 (IQR 16–31). Most patients underwent orthopedic surgery (52.8%) followed by intra-abdominal (28%) and bariatric surgery (19.2%). The majority received general anesthesia (83 patients, 66.4%). The median first 24-hour opioid consumption/kg was 1.3 mg/kg of an oral morphine equivalent dose (IQR 0.5–2.2 mg/kg). Twelve patients (9.6%) received ketamine infusion postoperatively. Three (2.4%) patients experienced unplanned ICU admissions. There was no mortality within 30 days of surgery.

**Table 1:** Patient characteristics.

Description	n = 125
**Demographic**	
Age (years)	59.7 ± 15.4
Male sex	46 (34.4%)
Weight (kg)	90.5 ± 23.7
**Medical history**	
High OSA risk^a^	33 (26.4%)
Lung disease	23 (18.4%)
Cardiac disease	9 (7.2%)
**Risk stratification**	
ARISCAT	19 (16, 31)
ASA	2 (2, 3)
**Surgical details**	
Orthopedic	66 (52.8%)
Abdominal	35 (28.0%)
Bariatric	24 (19.2%)
Duration of Surgery (min)	130 (103, 161)
**Anesthesia and analgesia**	
Any general anesthesia	83 (66.4%)
Regional anesthesia	42 (33.6%)
Duration of stay in PACU (min)	60 (41.3, 87.3)
24-hour opioid/kg (OMEDD/kg)	1.3 (0.5, 2.2)
Postoperative ketamine	12 (9.6%)
**Postoperative course**	
ICU admissions within seven days	3 (2.4%)
Length of stay (days)	4 (3, 5)
30-day mortality	0 (0%)

The results are presented as mean ± standard deviation, median (IQR), or number (%). OSA = obstructive sleep apnea; ASA = American Society of Anesthesiologists physical status; ARISCAT = Assess Respiratory Risk in Surgical Patients in Catalonia; PACU = post-anesthesia care unit; OMEDD = oral morphine equivalent daily dose; ICU = intensive care unit.

^a^ High OSA risk means either untreated OSA or high probability of diagnosed OSA as determined by STOP-BANG ≥4 [31].

### Primary outcome

#### PAEs

**Fig 2:**
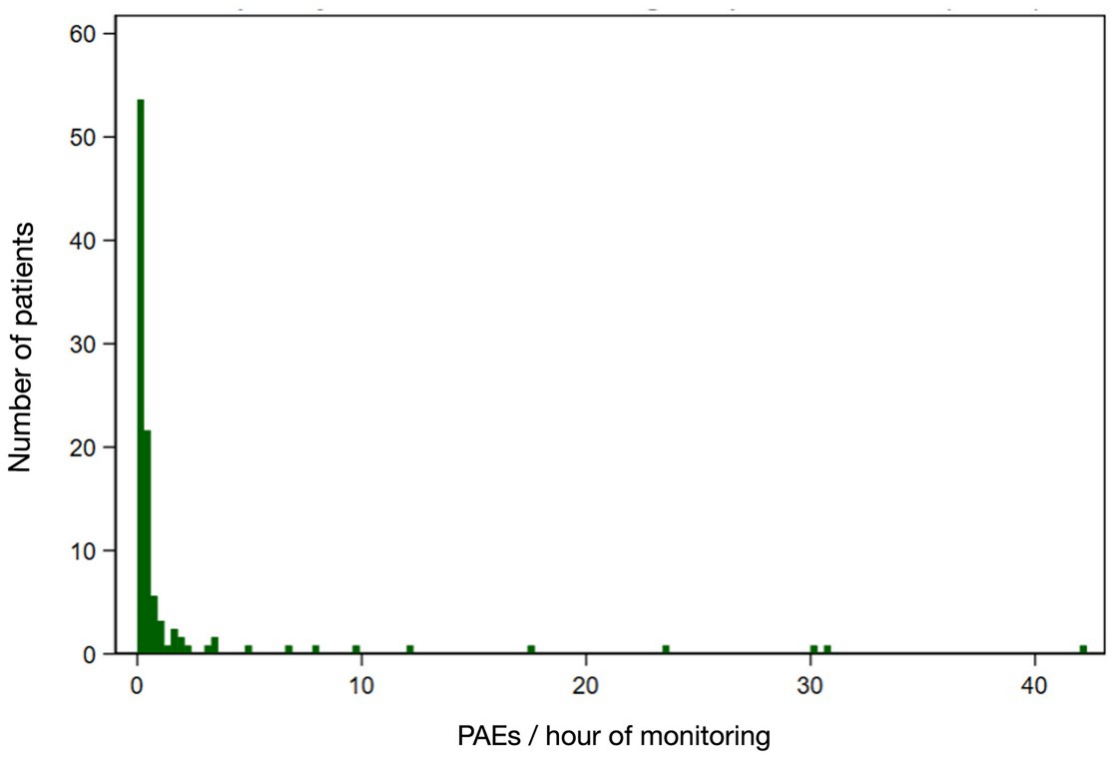
The frequency distribution of PAEs per hour of monitoring. The histogram shows the frequency of PAEs in an average hour of monitoring. 58 (46.4%) patients had 0 events/hour. The highest quartile of patients experienced 0.5–42.2 events/hour. PAEs = prolonged apnea events, defined as the number of times during the monitoring period of 2200 to 0800 after the first postoperative night that EtCO_2_ ≤5 mmHg for 30–120 seconds or EtCO_2_ ≤5 mmHg for >120 seconds in association with SpO_2_ ≤ 85%.

A total of 1800 PAEs were recorded among 67/125 (53.4%) patients on the first postoperative night (Table 2), of which 1769 lasted 30–120 seconds, and 31 lasted >120 seconds, associated with SpO_2_ < 85%. The frequency distribution of PAEs was positively skewed (Fig 2). Of the patients who experienced at least one PAE during the entire monitoring period (n = 67), the median (IQR) PAE per patient was four (2–12) (*cf*. for the entire cohort (n = 125), median (IQR) PAE per patient was 1 (0–4.5)). Expressed differently, on average, the median (IQR) PAE per hour for patients with at least one PAE was 0.5 (0.3–1.5) (*cf*. for the entire cohort (n = 125), median (IQR) PAE per hour was 0.1 (0–0.5)). Among patients with at least one PAE, the highest quartile (16 /67 patients) experienced a disproportionate number of PAEs (1603 PAEs, 89.1% of total).

**Table 2:** Descriptive statistics for PAEs and PPCs.

Description	Value
**PAE**	
Number of PAEs	1800
Number of patients with ≥1 PAE	67 (53.4%)
PAEs/patient	1 (0–4.5) [Max 336]
PAEs/hour	0.1 (0–0.5) [Max 42.2]
**PPC**	
Number of PPCs	54
Number of patients with ≥1 PPC	41
PPCs/patient	0 (0–1) [Max 3]
PPC type: Prolonged oxygen requirement	38 (30.4%)
PPC type: Atelectasis	7 (5.6%)
PPC type: Pleural effusion	7 (5.6%)
PPC type: Bronchospasm	1 (0.8%)
PPC type: Pneumonia	1 (0.8%)

The results are presented as either median (IQR) [max] or number (%), unless otherwise indicated. PAEs = prolonged apneic events (see text for definition). PPCs = postoperative pulmonary complications, as defined by Canet et al. [25]

### Secondary outcomes

#### AI, ODI, IPI, and SpO_2_

**Fig 3:**
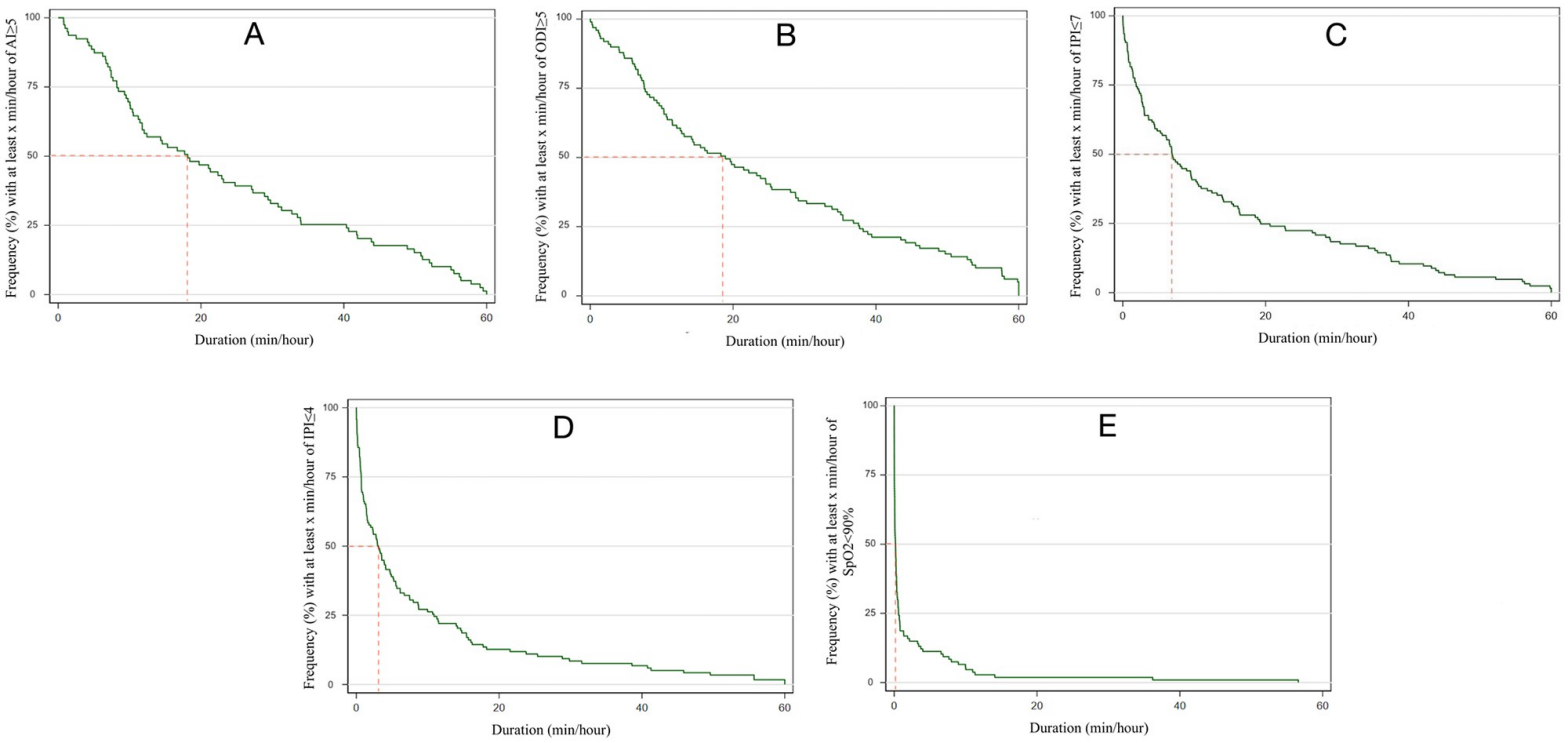
Frequency distribution of the proportion of recorded time with deranged AI, ODI, IPI, and SpO_2_ expressed as average minutes per hour of monitoring. **A)** Frequency distribution of the proportion of recorded time with AI ≥ 5. **B)** Frequency distribution of the proportion of recorded time with ODI ≥ 5. **C)** Frequency distribution of the proportion of recorded time with IPI ≤ 7. **D)** Frequency distribution of the proportion of recorded time with IPI ≤ 4. **E)** Frequency distribution of the proportion of recorded time with SpO_2_ < 90%. The red line represents the points at which half the population experience derangements in AI, ODI, IPI, or SpO_2_. AI = apnea index; the average number of apnea events (absence of respiratory activity for ≥10 seconds) per hour. ODI = oxygen desaturation index, the number of times that oxygen saturation drops by ≥4% from baseline within a 240 second period. IPI = integrated pulmonary index, a global assessment of cardiorespiratory status incorporating all available monitoring results from the Capnostream monitor, with 10 reflecting normal physiology and 1 being the most deranged.

The proportion of recorded time of deranged AI, ODI, IPI, and SpO_2_ is described in Fig 3 and is expressed as average minutes per hour of monitoring. AI and ODI were deranged (i.e., ≥5 events/hour) for every hour of monitoring for up to 18.1 minutes and 18.9 minutes, respectively, in at least half of all patients (Fig 3A and 3B). The duration of deranged IPI that met the threshold for requiring attention (IPI ≤7) was up to 6.9 minutes for every hour of monitoring in at least half of all patients (Fig 3C). The duration of deranged IPI that met the threshold for requiring intervention (IPI ≤4) was up to 3.1 minutes for every hour of monitoring in at least half of all patients (Fig 3D). However, for every hour of monitoring, deranged SpO_2_ (SpO_2_ <90%) was typically brief, occurring for <1 minute for the majority (82.2%) of patients (Fig 3E). Twelve (9.6%) patients suffered ≥5 minutes per hour of deranged SpO_2_.

#### PPCs

Forty-one (32.8%) patients experienced PPCs (Table 2). Prolonged oxygen requirement was the most common PPC suffered (70.3% of all PPCs). Eleven (8.8%) patients experienced more than one PPC. Of the patients who experienced at least one PPC (n = 41), 93% required prolonged oxygen therapy, 17% developed atelectasis, 17% developed pleural effusions, 2.4% developed bronchospasm, and 2.4% developed pneumonia.

#### Multivariable analysis

**Fig 4:**
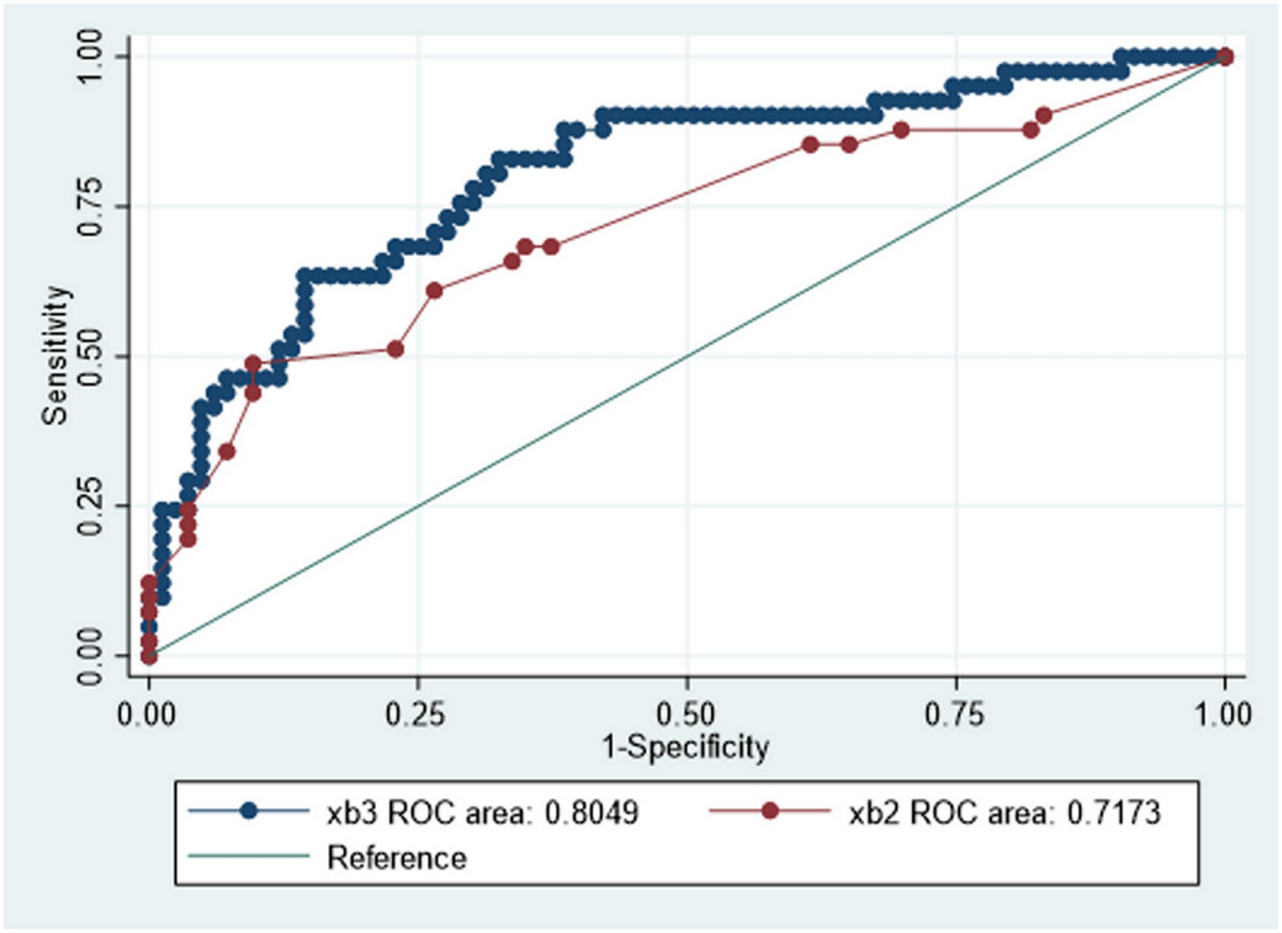
Comparison of discriminatory performance using ARISCAT vs. Model 1 for predicting PPCs. The red line represents the ROC for ARISCAT. The dark blue line represents the ROC for Model 1. The light blue line represents the reference line. Model 1 was derived using multivariable analysis incorporating the ARISCAT score, age, and 24-hour opioid consumption/kg. AUROC for ARISCAT was 0.717 (95% CI 0.616–0.819), and AUROC for Model 1 was 0.805 (95% CI 0.721–0.889). Comparisons between the two ROC curves were statistically significant (p = 0.016). ARISCAT = Assess Respiratory Risk in Surgical Patients in Catalonia. PPCs = postoperative pulmonary complications, as defined by Canet et al. [25]; ROC = receiver operating characteristic; AUROC = area under receiver operating curve.

Variables that were provisionally noted and incorporated into the multivariable regression analysis are provided in Table 3. This initial binary logistic regression identified nine factors associated with PPCs with a *p*-value <0.20, which were entered into the multivariable model. However, in the final multivariable model, only age (OR = 1.05, 95% CI 1.02–1.09, p = 0.002), 24-hour opioid consumption/kg (OR = 1.55, 95% CI 1.08–2.23, p = 0.019), and ARISCAT score (OR = 1.07, 95% CI 1.03–1.12, p = 0.001) were significantly associated with PPCs (Table 4). The multivariable model (Model 1) showed good predictive ability with are under receiver operative curve (AUROC) of 0.805 and was well-fitted (Hosmer–Lemeshow chi-squared = 12.08, p = 0.15). Compared with using ARISCAT alone, Model 1 improved the predictivity ability for discriminating PPCs (AUROC for ARISCAT alone = 0.717 *c/f* AUROC for Model 1 = 0.805, p = 0.016, as shown in Fig 4).

**Table 3:** Variables considered in the multivariable logistic regression analysis for PPCs.

Variable	Unadjusted OR (95% CI)	*p*-value	Adjusted OR (95% CI)	*p*-value
Age	1.05 (1.02–1.08)	0.007	1.06 (1.01–1.10)	0.009
Lung disease	2.2 (0.87–5.53)	0.096	1.71 (0.48–6.03)	0.406
High OSA risk	2.61 (1.03–6.58)	0.058	1.69 (0.42–6.79)	0.618
ARISCAT score	1.07 (1.03–1.11)	<0.001	1.10 (1.01–1.19)	0.024
Duration surgery	1.01 (1.00–1.02)	0.046	0.99 (0.98–1.01)	0.409
24-hr OMEDD/kg	1.78 (1.23–2.57)	0.007	1.50 (0.99–2.27)	0.055
Surgical risk group	3.47 (1.59–7.57)	0.001	0.76 (0.16–3.69)	0.737
Median IPI	0.90 (0.77–1.04)	0.161	1.05 (0.84–1.32)	0.651
Duration SpO_2_ <90%	1.00 (1.00–1.00	0.018	1.00 (1.00–1.00)	0.214

PPCs = postoperative pulmonary complications, as defined by Canet et al. [25]; OR = odds ratio; CI = confidence interval; OSA = obstructive sleep apnea; ASA = American Society of Anesthesiologists physical status; ARISCAT = Assess Respiratory Risk in Surgical Patients in Catalonia [25]; OMEDD = oral morphine equivalent daily dose; IPI = Integrated Pulmonary Index [24].

**Table 4:** Final multivariable logistic regression model for PPCs.

Variable	Odds Ratio	95% confidence interval	*p*-value
Age	1.05	1.02–1.09	0.002
24-hour OMEDD/kg	1.55	1.08–2.23	0.019
ARISCAT	1.07	1.03–1.12	0.001

OMEDD = oral morphine equivalent daily dose (normalized to weight); ARISCAT = Assess Respiratory Risk in Surgical Patients in Catalonia [25].

## Discussion

### Key findings

In this blinded observational study, we demonstrated that at least one PAE occurred in more than half the monitored low-risk patients on the first postoperative night after undergoing major surgery. The duration of deranged AI (≥5) and ODI (≥5) was up to 30% for each hour of monitoring in at least half of all patients. The duration of deranged IPI requiring attention (i.e. IPI ≤7) lasted for up to 12% of each hour of monitoring in at least half of all patients, while the duration of deranged IPI requiring intervention (i.e. IPI ≤4) lasted for up to 5% of each hour of monitoring in at least half of all patients. Nevertheless, the duration of significant hypoxia was uncommon. Despite the inclusion of a low-risk cohort, as evidenced by the ARISCAT score, the few unplanned ICU admissions and low 30-day mortality, PPCs were common, occurring in approximately one-third of patients. No variables that improved the discriminatory power of ARISCAT score for detecting PPCs were detected by blinded monitoring in the exploratory modeling. However, using age and the first 24-hour opioid consumption/kg improved the model.

### Relationship to other studies

Several large studies have investigated postoperative respiratory events using capnography. In a large multicenter observation study, Khanna et al. reported that 44% of patients suffered at least one respiratory depression event in the first 48 hours postoperatively [15]. Overdyk et al. noted widespread bradypnea (respiratory rate <10 breaths/min) in 41% of postoperative patients receiving parenteral opioids. In contrast, McCarter et al. noted a much lower rate of bradypnea (1.4%) in postoperative patients receiving PCA, but the authors used a lower respiratory rate to define bradypnea (respiratory rate <6 breaths/min [32]). Weiniger et al. reported on “apnea alert events,” which the authors defined similarly to our definition of PAEs. The authors found these events occurred in 53% of women receiving low intrathecal morphine for caesarian section delivery, which correlates with the findings of the present study.

In contrast with previous continuous oximetry studies, in our cohort, hypoxia was relatively uncommon. Overdyk et al. found that 12% of postoperative patients had hypoxia (SpO2 < 90%) for at least 3 minutes [18]. Sun et al. reported a 21% incidence in SpO2 < 90% for at least 10 minutes/hour of monitoring [11]. However, in both these studies, the patients did not routinely receive supplemental oxygen, which was mandated by local policy for the use of PCA at our institution. This could have masked the potential for hypoxia in our study. In addition, Sun et al. monitored their patients for a longer period (up to 72 hours), allowing for a greater window of time during which hypoxia could manifest.

PPCs were common in our cohort of patients, and our results are in keeping with the rates reported in the literature [2, 25, 33, 34]. Most PPCs were mild, consisting of a prolonged oxygen requirement and atelectasis. Nonetheless, our findings are important, as even mild PPCs have been associated with increased mortality, increased hospital length of stay, and resource utilization [2, 6, 25].

Although only an exploratory result, the significant association between 24-hour opioid consumption/kg and PPCs after adjusting for the ARISCAT score was interesting. High use of preoperative opioids has been associated with PPCs [35]. Nevertheless, the relationship between opioids and PPCs is complex due to the concurrent alteration of respiratory mechanics by anesthesia and surgery. Atelectasis ensues rapidly after induction of anesthesia, and mechanical ventilation and can persist for several days postoperatively [7]. Sputum clearance by mucociliary action is impaired by tracheal intubation. Hypnotics and analgesic agents interfere with the central respiratory drive by increasing hypercapnia and the hypoxic threshold. Direct disruption of the respiratory muscle or pain from surgical incision can lead to ineffective ventilation and cough. The combination of these effects can predispose a patient to more significant PPCs, such as pneumonia and respiratory failure [6]. A higher opioid requirement can also be a marker for postoperative pain, which contributes to inadequate ventilation and its negative ramifications. Better pain control, such as epidural analgesia for abdominal surgery, has been shown to reduce PPCs [36, 37].

Opioids can also lead to sleep-disordered breathing (SDB), characterized by apneas and hypopneas. Using portable polysomnographic monitoring, Chung et al. demonstrated an association between opioids and postoperative SDB [14], which could occur even in patients without prior polysomnographic evidence of OSA. However, if the patient had prior OSA, SDB can worsen to a greater degree in the postoperative period [10]. In our study, at least 26.4% of patients had untreated OSA or were at high-risk of undiagnosed OSA. Many also had elevated body weight suggestive of obesity. Obesity impairs respiratory mechanics and is closely associated with OSA [38, 39]. OSA increases sensitivity to opioid and increases the risk of PPCs and other critical postoperative respiratory events [8, 40, 41]. Through potentiating SDB in the susceptible patients, opioid use may be associated with PPCs.

The use of continuous capnography with pulse oximetry is not equivalent to an attended polysomnography in a sleep laboratory, which is the gold standard in studies on sleep-disordered breathing. Numerous sophisticated physiological, mechanical, and electrical parameters have been measured in such studies [37], which require the interpretation by a clinician. Nevertheless, devices that measure continuous noninvasive capnography with pulse oximetry can combine relatively few parameters—namely, EtCO_2_, SpO_2_, and the respiratory rate—to provide information on potential sleep-disordered events, such as apneic or oxygen desaturation events. However, these simple devices are not considered adequate for home sleep apnea testing [42], even if they report similar-sounding indices.

Continuous respiratory monitoring could also reduce morbidity from opioid-related respiratory depression in standard care wards by offering an early warning system and opportunity for intervention [17, 20]. The first 24 hours following surgery are a particularly high-risk period for opioid-related respiratory depression due to high opioid requirements [5, 8, 9]. Capnography offers sensitive continuous monitoring of respiratory rate, which is often the first vital sign to become abnormal in an opioid-related respiratory depression event [43]. Implementing continuous capnography has been shown to reduce activation of rapid response teams and to reduce transfers to a higher-level care ward [21, 22]. However, whether it improves patient outcomes overall remains to be seen [20].

### Implications and future directions

Using continuous oximetry and capnography monitoring detected frequent respiratory aberrations in a low-risk major surgical patient cohort that would not normally receive close monitoring. PPCs were common among this cohort; however, they did not appear to be associated with PAEs or deranged physiological indices detected by continuous oximetry and capnography after accounting for other variables.

Further work is required to determine the threshold of monitoring aberrancies that would enable continuous oximetry and capnography to be used with appropriate sensitivity and specificity. For example, the IPI level that breached the threshold for requiring intervention was found to occur for up to 3.1 minutes/hour in more than half of all monitored patients. This could be an appropriately sensitive threshold to trigger action. However, the specificity of this threshold to detect clinically relevant outcomes, such as PPCs, is uncertain. Future work should be extended to include a higher-risk surgical population. Furthermore, given the frequency of aberrations detected, research is required to determine the effect of alarm fatigue on cognitive load and clinical behavior. Potential ways of mitigating alarm fatigue may be to utilize such monitors only in a cohort with high predictive probability of requiring intervention (i.e. a high-risk cohort) [44]; to allow for more significantly deranged values before alarms are triggered [19]; or to develop more advanced algorithms to determine risk, inclusive of the use of machine learning [45].

### Strengths

Our study had several strengths. We included a relatively large number of subjects at low risk of postoperative morbidity, despite having major surgery. Therefore, we are confident that the results of the present study have applicability in other settings involving similar cohorts of patients undergoing similar types of operations. Of note, many patients having major surgery do not undergo continuous monitoring on the first postoperative night due to the limited resource of high-dependency units or ICUs.

We chose a robust definition of PAEs, requiring a fall in SpO_2_ if prolonged, with low EtCO_2_ and not necessarily a value of zero to define apnea. These metrics were guided by the literature [15, 46].

Another strength of our study was the blinded methodology, which allowed us to investigate the effects of monitoring in the absence of a change in behavior from frequent alarms or the effect of close monitoring itself.

### Limitations

Our methodology was subject to user error. This included the possibility of the dislodgement of monitoring devices, which would only be corrected during routine intermittent nursing checks. However, our methodology provides an insight into the frequency of aberrations in monitoring detected by this machine when used in real-world conditions, which has not been adequately captured in other non-blinded studies.

Of note, we only included patients for whom we had complete data in the analysis. Therefore, no comment can be made on whether those patients who were not included in the analysis would have increased or decreased the frequency of PAEs or any other aberrations in monitoring. This could represent a source of bias. Nevertheless, our aim was to capture the frequency of monitoring aberrancies in a low-risk cohort undergoing major surgery, and this was achieved with our methodology.

## Conclusions

At least one PAE occurred in more than half of all patients on the first postoperative night after major surgery in a low-risk cohort when using the Capnostream^™^ monitor to detect continuous oximetry and capnography. Aberrations in the displayed AI and ODI were present for a significant portion of every hour of monitoring, while the monitor warned of cardiorespiratory status requiring intervention (i.e. IPI≤4) for up to 5% of every hour of monitoring in more than half of all patients. Nevertheless, significant hypoxia was uncommon, which may demonstrate the benefit of mandatory oxygen supplementation for patients receiving PCA. PPCs were common in this cohort, but in multivariable modeling, no variables detected by the Capnostream were associated with PPCs. Future research should address the significance of continuous monitoring in higher-risk surgical groups and clarify the clinical significance of the detected aberrations.

## Supporting information

S1 Appendix Data dictionary—Definitions and type of data. (DOCX)

S1 Data (XLSX)
