# Supplementary material for: Nocturnal respiratory abnormalities among ward-level postoperative patients as detected by the Capnostream 20p monitor: A blinded observational study
Source: PLoS One. 2023 Jan 20;18(1):e0280436. doi: 10.1371/journal.pone.0280436 (PMC9858304; doi:10.1371/journal.pone.0280436)
Supplement: S1 Appendix — (DOCX) [file pone.0280436.s001.docx]

# **Appendix 1 – Data dictionary – definitions and type of data.**

- Age, expressed in years, determined at time of surgery. Continuous data.*
- Cardiac disease, defined as the presence of either ischemic heart disease, left or right ventricular heart failure or diastolic dysfunction. Categorical data.
- Lung disease, defined as presence of either asthma, chronic airway disease, bronchiectasis or interstitial lung disease. Categorical data.*
- High OSA risk, defined as STOP-BANG >3 or known and untreated OSA. Ordinal data.*
- ASA physical status, assessed by two independent anesthesiologist (CT, RH). Ordinal data.*
- ARISCAT score, used as a continuous variable and calculated as per Canet J, Gallart L, Gomar C, Paluzie G, Vallès J, Castillo J, et al. Prediction of postoperative pulmonary complications in a population-based surgical cohort. Anesthesiology. 2010;113(6):1338-50. Continuous data.*
- Regional anesthesia, defined as the use of any regional anesthesia including spinal anesthseia +/- sedation as the sole anesthetic technique. Categorical data.*
- Any general anesthesia, defined as occurring where regional anesthesia was NOT employed as sole technique. Categorical data.
- Duration of surgery, expressed in minutes, defined as time of entry to time of leaving operating room as recorded on electronic medical records. Continuous data.*
- Duration of stay in PACU (post-anesthesia care unit), expressed in minutes, defined as time of entry to tie of leaving PACU as recorded recorded on electronic medical records. Continuous data.
- 24-hour opioid consumption (OMEDD/kg) = Oral Morphine Equivalent Daily Dose (in milligrams) per kg of weight in the first 24 hours after surgery, beginning when the patient entered postanesthesia care unit. Conversion to OMEDD was based on tables provided in Australian Institute of Health and Welfare 2018. Opioid harm in Australia and comparisons between Australia and Canada. Cat. No. HSE 210. Canberra: AIHW. Continuous data.*
- Postoperative ketamine, defined as any infusion of ketamine for the purpose of analgesia within 7 days of surgery. Categorical data.
- High surgical risk group, defined as having an incision that involves the upper intestinal or intrathoracic region. Categorical data.*
- Median PAE / hour, expressed as count per hour, defined as either 1) EtCO2 ≤ 5 mmHg for 30 – 120 seconds, or 2) EtCO2 ≤ 5 mmHg for > 120 seconds in association with SpO2 ≤ 85%, as captured by the Capnostream monitor. Continuous data.*
- Median AI / hour, expressed as count per hour, defined as the median displayed AI/hour during monitoring as captured by the Capnostream monitor. Continuous data.*
- Median ODI / hour, expressed as count per hour, defined as the median displayed ODI/hour during monitoring as captured by the Capnostream monitor. Continuous data.*
- Median IPI / hour, expressed as count per hour, defined as the median displayed IPI/hour during monitoring as captured by the Capnostream monitor. Continuous data.*
- Duration of SpO_2_ <90%, expressed in seconds, defined as the duration that displayed pulse oximetry values were <90%, as captured by the Capnostream monitor. Continuous data.*
- Duration that Respiratory Rate ≤7, expressed in seconds, defined as the duration that displayed respiratory rate was ≤7, as captured by the Capnostream monitor. Continuous data.*
- Duration of Respiratory Rate ≥26, expressed in seconds, defined as the duration that displayed respiratory rate was ≥26 as captured by the Capnostream monitor. Continuous data.*

* Factors considered in regression modelling.
